# Construction of a genetic sexing strain for *Aedes albopictus*: a promising tool for the development of sterilizing insect control strategies targeting the tiger mosquito

**DOI:** 10.1186/s13071-018-3212-y

**Published:** 2018-12-24

**Authors:** Cyrille Lebon, Aude Benlali, Célestine Atyame, Patrick Mavingui, Pablo Tortosa

**Affiliations:** 1 Groupement d’Intérêt Public Cyclotron Océan Indien (CYROI), 2 rue maxime Rivière, 97490 Ste Clotilde, Ste Clotilde, France; 2 Symbiosis Technologies for Insect Control (SymbioTIC). Plateforme de Recherche Cyroi, 2 rue Maxime Rivière, 97490 Ste Clotilde, Ste Clotilde, France; 3 Université de La Réunion, Unité Mixte de Recherche Processus Infectieux en Milieu Insulaire Tropical (UMR PIMIT). CNRS 9192, INSERM 1187, IRD 249. Plateforme de recherche CYROI, 2 rue Maxime Rivière, 97490 Ste Clotilde, La Réunion, France; 4 Université de Lyon, Lyon, France, Université Lyon 1, Villeurbanne, France; CNRS, UMR 5557, Ecologie Microbienne, Villeurbanne, France, INRA, UMR1418, Villeurbanne, France

**Keywords:** *Aedes albopictus*, Genetic Sexing Strain, sex separation, Sterile Insect Technique, Incompatible Insect Technique

## Abstract

**Background:**

*Aedes albopictus* is an invasive mosquito species of global medical concern as its distribution has recently expanded to Africa, the Americas and Europe. In the absence of prophylaxis protecting human populations from emerging arboviruses transmitted by this mosquito species, the most straightforward control measures rely on the suppression or manipulation of vector natural populations. A number of environmental-friendly methods using mass releases of sterilizing males are currently under development. However, these strategies are still lacking an efficient sexing method required for production of males at an industrial scale.

**Results:**

We present the first Genetic Sexing Strain (GSS) in *Ae. albopictus*, hereafter referred as Tikok, obtained by sex linkage of the *rdl* gene conferring dieldrin resistance. Hatch rate, larval survival and sex ratio were followed during twelve generations. The use of dieldrin at the third larval stage allowed selecting 98 % of males on average.

**Conclusion:**

A good production rate of Tikok males makes this GSS suitable for any control method based on mass production of *Ae. albopictus* males*.* Despite limitations resulting from reduced egg hatch as well as the nature of the used insecticide, the construction of this GSS paves the way for industrial sex separation of *Ae. albopictus*.

## Introduction

The recent Zika virus (ZIKV) epidemic has put vector borne diseases under the spotlight, and further confirmed the growing concern of emerging vector-borne pathogens [1]. This epidemic shows similarities with chikungunya virus (CHIKV) emergence a decade ago: both CHIKV (alphavirus) and ZIKV (flavivirus) were initially known to cause outbreaks limited in space and time before expanding at a global scale. These epidemics tend to remind that we will most certainly experience future epidemics caused by arboviruses mostly unknown to the scientific community. As a consequence, the prophylactic treatments needed for the control of such diseases will be either scarce or absent.

One way to prevent the spread of emerging arthropod-borne pathogens is to limit their transmission through the control of their invertebrate vectors. Such control methods, which have been so far mostly implemented through the use of synthetic pesticides, have proven efficient and notably allowed eradicating malaria from several territories worldwide following World War II. However, the recurrent selection of insecticide resistance in vector natural populations [2], together with unwanted effects on non-target species have led to a ban of several synthetic pesticides and in turn stimulated the development of a handful of environmentally friendly methods. The field of innovative vector control development is currently exceptionally dynamic [3], and several ongoing programs have reached the step of field pilot trial assays [4–6]. Among the strategies aiming at suppressing mosquito natural populations, sterilizing technologies based on the mass release of sterile/incompatible males have been experiencing a spectacular revival. These technologies use either Gamma- or X- ray irradiation [7], transgenesis [8], *Wolbachia* symbiotic bacteria [9] or more sophisticated associations such as the recently proposed boosted Sterile Insect Technique [10], in which males are sterilized through irradiation and then used as dispersers of entomopathogenic viruses and/or insecticides [11]. All these technologies rely on the availability of an efficient sexing method allowing the separation of males and females at industrial scales, which is compulsory for mass release of treated males.

The control of vector populations through the release of sterile males has proven successful for pest species such as *Cochliomyia hominivorax* or *Ceratitis capitata* [12, 13]. By contrast, these vector control strategies have not been implemented at industrial scales for the control of mosquitoes. The availability of an efficient sexing method is indeed one of the main technological locks. Thus far, pilot trials have been using manual or mechanical separation techniques, which are not either up scalable or fully satisfactory [14]. Indeed, mechanical separation relies on sexual dimorphism, which can allow efficient sieving using glass plate size sorters as reported for *Ae. aegypti* [8]. However, sexual dimorphism may not be sharp enough in some species, such as *Ae. albopictus*, for which sieving is notoriously challenging and results in important reductions in the recovery of male pupae [15, 16].

Among the several methods currently explored for sex separation in *Ae. albopictus* [17], we used a dieldrin resistance conferring marker to construct a Genetic Sexing Strain (GSS), essentially adapting a strategy previously used for *Culex tarsalis* [18] and *Anopheles arabiensis* [19]. Sex linkage of the *rdl* gene, conferring dieldrin resistance, was obtained through X-ray irradiation and selection of the sex biased mosquito lines. The data presented herein provide, to our knowledge, the first GSS reported to date for *Ae. albopictus*, and hereafter referred as Tikok. We present the performance and some life history traits of Tikok and discuss future improvements that will accelerate the implementation of sterilizing strategies targeting the tiger mosquito.

## Methods

### Mosquito stocks and rearing

Two *Ae. albopictus* lines originally sampled in two localities on La Réunion Island (Plaine des Palmistes and La Providence) were selected for this study. These localities correspond to an elevated rural area and a coastal urban park of La Réunion Island sheltering natural mosquito populations displaying low and high *rdl*^*R*^ allelic frequencies, respectively [20]. Larvae were reared at a density of approximately 1000 first instar larvae in white plastic trays (30 × 40 cm with a depth of 6 cm) containing 3 l of distilled water and were fed *ad libitum* with a mixture of rabbit- (Naturalima, Urcoopa, Saint Paul, Réunion) and fish-food (Tetra GmbH, TetraMin, Heinsberg, Germany). Upon pupation, males and females were separated manually under a binocular loop using terminalia dimorphism and placed into insect rearing cages (30 × 30 × 30 cm) until emergence. Adults were fed with 10 % sucrose solution (w/v) and females were blood-fed using the Hemotek feeding system (Discovery Workshops, Lancashire, UK) and defibrinated cow blood provided by the regional slaughterhouse. Such blood-feeding does not require ethical clearance.

### Determination of the dieldrin diagnostic dose

**Fig. 1:**
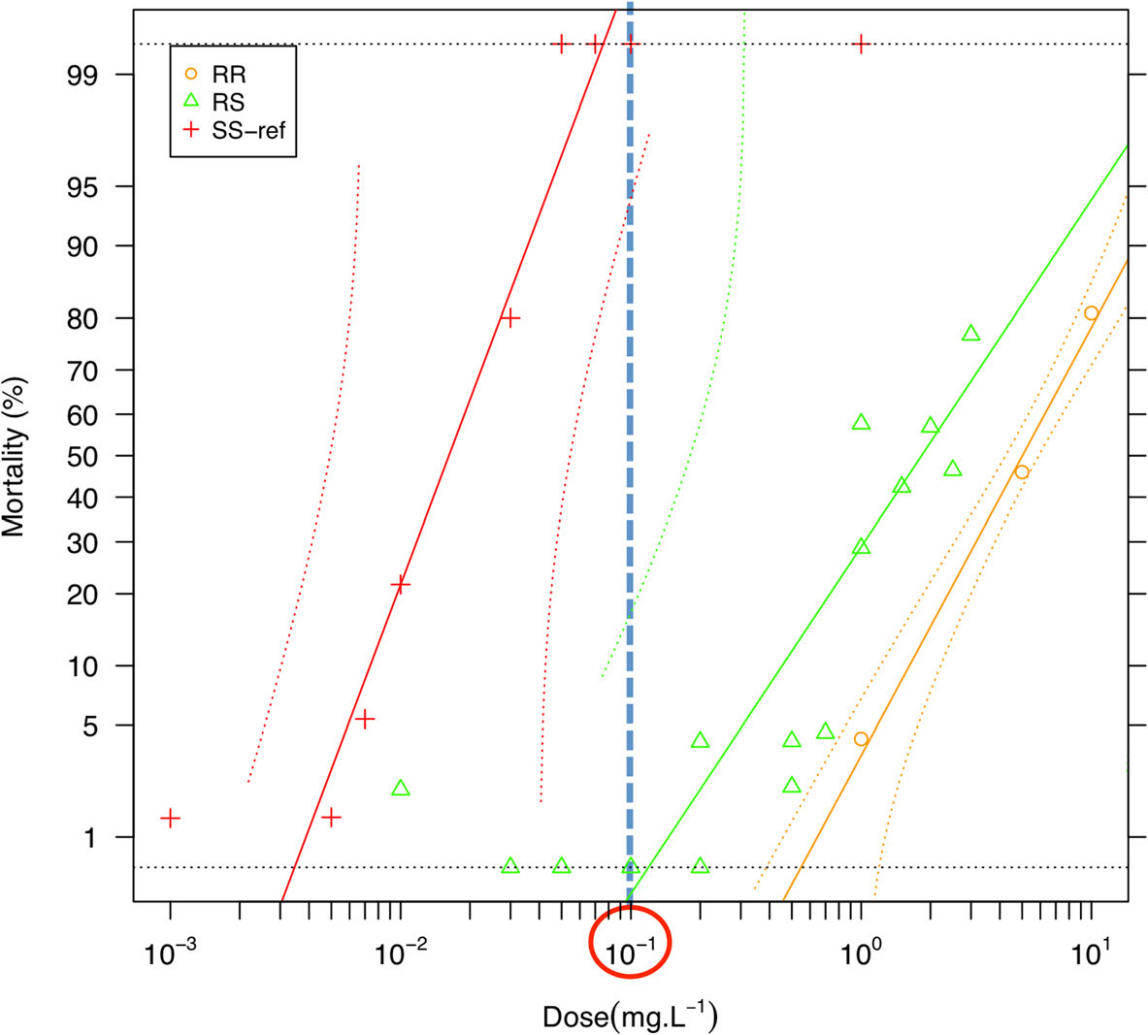
Survival of R-Run (RR), S-Run (SS) and heterozygous (RS) third instar larvae following 24 h treatment with increasing dieldrin concentrations. The diagnostic dose is circled and corresponds to the dieldrin dose for which all sensitive mosquitoes die while all heterozygous mosquitoes survive

A 1000 ppm dieldrin (Dr Ehrenstorfer, Germany) stock solution was prepared in absolute ethanol and used as a stock for further dilutions. Batches of 25 third-instar larvae were placed in 100 ml of water containing 0 to 10 ppm dieldrin. Three replicates were prepared for each insecticide concentration and cups were conserved at room temperature. All surviving and dead larvae were counted in each cup after 24 h and data were analysed using BioRssay 6.2 [21]. The diagnostic dose was determined by testing homozygous R-Run (RR) and S-Run lines, (SS) together with heterozygous F1 (RS) larvae obtained by crossing R-Run males with S-Run females. For each homozygous susceptible (SS), heterozygous (RS) or homozygous resistant (RR) mosquitoes and each dieldrin dose, three batches of 25 third-instar larvae were placed in 100 ml of water containing 0 to 10 ppm dieldrin, and left for 24 h at room temperature. Survival was plotted for each dieldrin dose and mosquito genotype. As shown in Fig. 1, a dieldrin concentration of 0.1 ppm killed 100 % of S-Run larvae but none of the heterozygous or R-Run larvae, and was thus selected as the diagnostic/selective dose used for all dieldrin selection steps.

### Molecular typing

Adult mosquito DNA was extracted as previously described [22]. A PCR-RFLP test [20] was used to detect the Ala to Ser substitution in *rdl* conferring dieldrin resistance in *Cx. pipiens* and *Ae. albopictus*. PCR was run for 30 cycles (94°C for 30 s, 52°C for 30 s, and 72°C for 1 min), followed by 5 min at 72°C. The PCR product was then digested for 3 h at 60°C with *BstAP*I restriction endonuclease (New England Biolabs, Evry, France), which selectively cleaves the susceptible allele [20]. Allelic profiles were then visualized using 2 % agarose electrophoresis of digested PCR product stained with GelRed.

### X-Ray irradiation and GSS identification

Chromosomal translocations were induced using a BloodXrad X-ray irradiator (Cegelec, Bretigny sur Orge, France) located at the Institut Français du Sang, Centre Hospitalier Universitaire de La Réunion. For this, 100 *rdl* homozygous resistant (R-Run) male pupae were placed in a jar (Ø: 11 cm; H: 8.5 cm) containing 40 ml distilled water and were irradiated with one of the two selected irradiation doses: 20 Gy and 25 Gy. Each of the two batches of males, corresponding to the two irradiation doses, were then crossed *en masse* with *rdl* homozygous susceptible (S-Run) females, leading to the F0 heterozygous generation. For each irradiation dose, 40 crosses (80 total) were performed using one F0 (heterozygous) male and five S-Run females into a small insect holding cage (12 × 12 × 12 cm). Eggs from each cage were allowed to hatch and up to 50 third-instar larvae were placed at room temperature for 24 h in plastic cups containing 100 ml of water supplemented with 0.1 ppm dieldrin. Sex ratio was determined at pupal stage and all broths displaying > 60 % males following dieldrin selection were allowed to emerge. Males from each progeny were then backcrossed with S-Run females and dieldrin pressure was maintained at each following backcross.

### Measurement of life history traits

Hatch rates as well as larvae survival rates and sex ratios following dieldrin treatment were measured for Tikok. For this, eggs were allowed to hatch for 24 h in closed cups containing 250 ml water supplemented with 1 ml of 7.5% rabbit food solution (Naturalima Urcoopa, Saint Paul, Reunion). Following hatching, filter papers were observed under a binocular microscope and hatched (opened) and unhatched (closed) eggs were counted. Larvae survival was measured by counting the total number of alive larvae before and after dieldrin selection (0.1 ppm, 24 h). We sexed mosquitoes at the pupal stage using a sexual dimorphism on Terminalia. Mean egg hatches were compared using the Student's t-test.

## Results

### Construction of dieldrin resistant (R-RUN) and susceptible (S-RUN) homozygous mosquito lines

The construction of a GSS through sex linkage of a selectable insecticide resistance gene requires the availability of homozygous resistant and susceptible mosquito lines. A dieldrin sensitive strain was constructed as follows: 20 cages were set with five females and one male in each cage. Adult mosquitoes were left for 48 h and each male was then genotyped at the *rdl* locus following a previously described procedure [20]. Only those cages whith homozygous *rdl*^*S*^ males were conserved and females were given a blood meal. Two days later, each female was placed in a separate cage and allowed to lay eggs, which were conserved while females were genoytyped. Those eggs laid by *rdl*^*S*^ homozygous females were poolled and immersed in water for hatching, providing an homozygous sensitive line hereafter named S-Run.

The construction of a homozygous resistant strain required three rounds of dieldrin selection to increase the frequency of *rdl*^*R*^ allele in the cages. For this, third- and fourth-instar larvae from La Providence were placed in plastic cups containing 50 ml of water with increasing dieldrin concentration. F0 larvae were placed in 0.05 ppm dieldrin for 24 h and surviving larvae were subsequently fed until emergence. Eggs laid by surviving F0 females were allowed to hatch and third and fourth instar larvae were placed again under 0.05 ppm dieldrin. At F2, larvae were placed under 0.1 ppm dieldrin, surviving larvae were allowed to moult and pupae were sex separated. Isofemale lines were constructed following the same exact procedure as that implemented for the construction of S-Run, and eggs resulting from copulations between homozygous *rdl*^*R*^ male and female mosquitoes were pooled. Hatching eggs led to the homozygous *rdl*^*R*^ mosquito line hereafter referred as R-Run.

### X-Ray irradiation, identification and amplification of Tikok

**Fig. 2:**
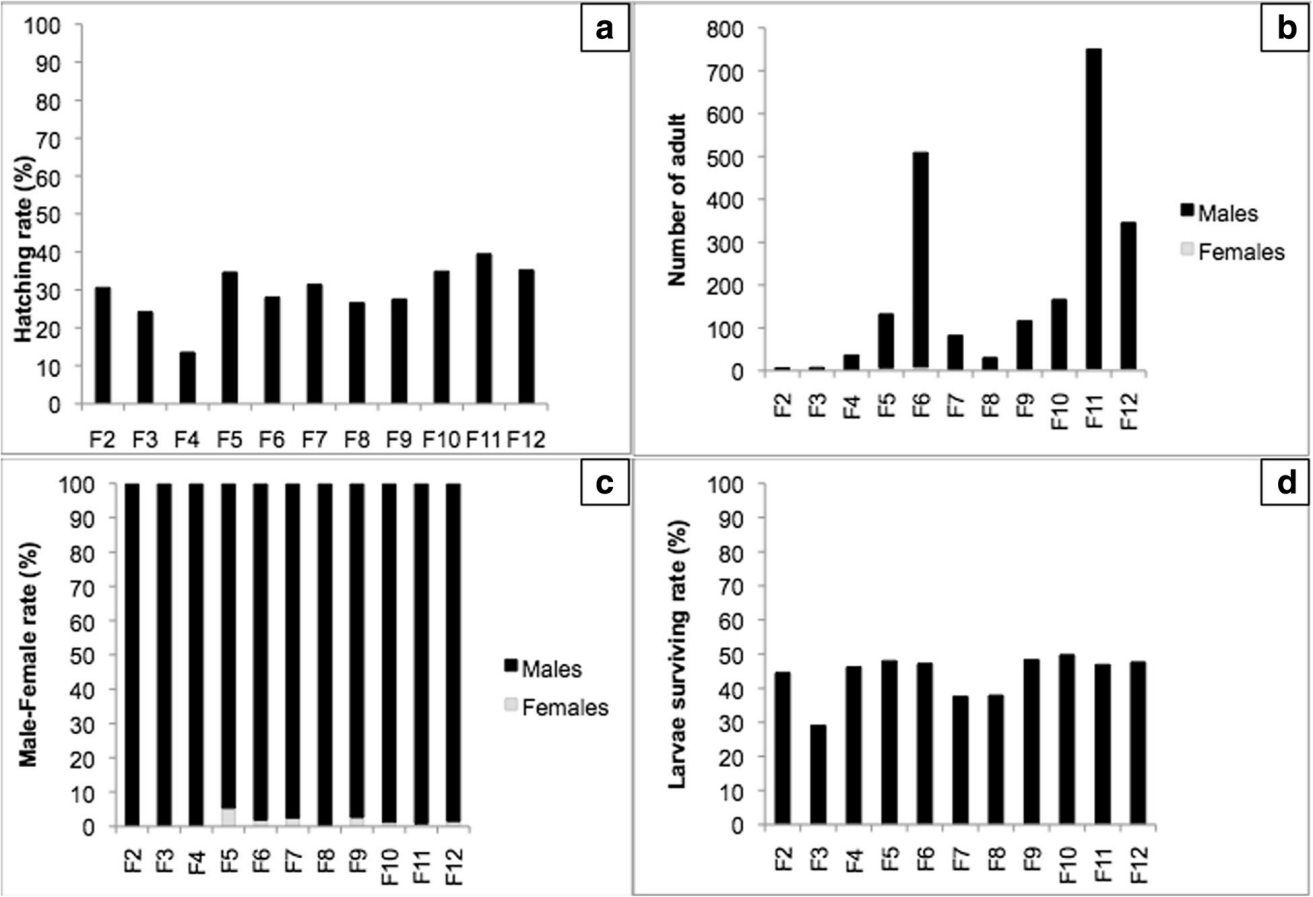
Tikok life history traits. The following parameters were collected over 12 generations under dieldrin pressure applied at each generation. The expected larvae survival rate is depicted as a hatched bar. *Abbreviations*: **a** hatch rate; **b** total number of surviving adults; **c** sex ratio; **d** larvae surviving rate

Following irradiation at 20 or 25 Gy, R-Run male pupae were allowed to emerge and then crossed *en masse* with virgin S-Run females for 48 h. Females were subsequently blood-fed and allowed to lay eggs. As expected, hatch rates were low for both irradiation doses (29.6 % and 11.3 % for 20 and 25 Gy irradiation, respectively). Following dieldrin selection, no difference was observed between these two irradiation doses either for larvae survival rate (20 Gy: 47.87 ± 1.19; 25 Gy: 49.61 ± 1.49) or sex ratio (20 Gy: 50.48 ± 1.52; 25 Gy: 54.70 ± 2.29). For each irradiation dose, 40 F1 dieldrin resistant males were individually crossed with S-Run females (one male and five females per cage), blood-fed and allowed to lay eggs. Hatch rates were significantly lower in crosses involving males irradiated at 25 Gy than in crosses involving males irradiated at 20 Gy (82.89 ± 3.33% and 93.38 ± 0.99%, respectively; Student's test, *t* = 3.53, *P* = 0.001). Only four males led to progenies exhibiting male biases exceeding 60 % and were further backcrossed. As shown on Table 1, a single male produced strong male bias (100 %) over 4 successive backcrosses. This mosquito line, hereafter referred as Tikok, was maintained and amplified while all other lines were discarded. At each generation, Tikok males were back-crossed with S-Run females with a 1:3 male/female ratio. Hatch and survival rates as well as sex ratio were measured for each generation. As shown in Fig. 2, hatch rate averaged 30 % over the entire experiment, larvae surviving rate following dieldrin treatment averaged 50 % and sex ratio bias was stable at 97.8 % (SD 5.96).

**Table 1:** *Aedes albopictus*, dieldrin selection of genetics sexing strain candidates. Egg hatch and sex ratios are provided for each candidate. Egg hatch is given for S-Run as a control (MD: missing data).

F0 Irradiation dose (Gy)	Generation	Starter male	Eggs number	Tikok Hatch rate (%)	S-Run Hatch rate (%)	Number of larvae exposed to dieldrin	Survival rate following dieldrin selection (%)	Number of male pupae	Number of pupae	Male %
20	F2	A	169	95.27	MD	138	48.55	40	66	60.61
20	F2	B	139	93.53	MD	122	51.64	37	56	66.07
25	F2	C	143	90.21	MD	114	41.23	24	33	72.73
25	F2	D	121	30.58	MD	18	44.44	6	6	100.00
20	F3	A	192	88.02	73.2	117	62.39	44	70	62.86
20	F3	B	352	86.93	73.2	251	51.79	64	116	55.17
25	F3	C	486	74.07	73.2	316	47.78	68	139	48.92
25	F3	D	149	24.16	73.2	31	29.03	7	7	100.00
20	F4	A	1242	25.93	87.1	284	50.00	62	141	43.97
25	F4	D	929	13.46	87.1	104	46.15	36	36	100.00
25	F5	D	1465	34.61	93.7	390	47.95	125	132	94.70
25	F6	D	6462	28.04	87.0	1540	47.14	500	509	98.23
25	F7	D	2288	31.42	79.8	538	37.55	80	82	97.56
25	F8	D	482	26.56	MD	95	37.89	30	30	100.00
25	F9_a	D	1218	32.02	89.5	317	48.90	102	115	88.70
25	F9_b	D	1747	27.48	89.5	429	48.25	113	116	97.41
25	F10	D	1499	34.89	91.7	350	49.71	164	166	98.80
25	F11	D	3241	39.46	MD	1648	46.84	744	750	99.20
25	F12	D	2461	35.19	86.6	759	47.56	340	345	98.55

### Fecundity of Tikok females obtained following insecticide pressure

Sex bias was not perfect and 2 % females descending from crosses between Tikok males and S-Run females survived the dieldrin treatment at the larval stage. Seven out of these surviving females were genotyped showing that they were all heterozygous at *rdl* (Table 2). This pattern suggests that these females actually resulted from meiotic recombination or translocation (see discussion) but not from treatment failure. Importantly, when these females were crossed with sensitive S-Run males, hatch rates were high (84.82 ± 6.91%) and sex ratio was not biased as dieldrin treatment of third instar larvae led to 50.33 % (± 18.12) males (see Table 2).

**Table 2:** Life history traits of larvae resulting from Tikok dieldrin resistant females crossed with S-Run males.

Generation	Female ID	Hatch rate (Total number of eggs)	Genotype	Number of larvae exposed to dieldrin	Survival rate following dieldrin selection (%)	Number of male pupae	Number of female pupae	Male %
F10	A	100.00 (4)	RS	NT	NT	NT	NT	NT
F10	B	98.90 (91)	RS	NT	NT	NT	NT	NT
F10	C	92.86 (14)	RS	NT	NT	NT	NT	NT
F11	D	48.28 (29)	RS	9	22.22	2	0	100.00
F11	E	74.31 109)	RS	58	48.28	14	14	50.00
F11	F	92.96 (71)	RS	39	38.46	2	12	14.29
F11	G	86.44 (59)	RS	52	53.85	10	17	37.04

### Stability of Tikok

In order to test a possible drift of Tikok in the absence of selective pressure, a part of layers was not treated with dieldrin after the 7th generation. For this, males and females were kept in rearing cages following emergence and the line was maintained without insecticide pressure during all subsequent generations. At each generation, eggs were recovered on two distinct filter papers. One was used to establish the next generation while the other was used to measure the level of dieldrine resistance following the standardized procedure detailed in the Materials and methods section and further discarded. Egg hatch steadily increased from 25.7 % to 92.7 % over 4 generations while larvae survival following dieldrin treatments decreased from 47.14 % to 3.20 % in the meantime. No resistant larvae were observed after 5 generations in the absence of insecticide pressure (Table 3).

**Table 3:** Tikok performance without dieldrin selection.

Generation	Egg number	Hatch rate (%)	Number of larvae exposed to dieldrin	Survival rate following dieldrin selection (%)	Number of male pupae	Number of pupae	Male %
F1	1219	25.68	280	47.14	70	70	100.00
F2	2147	40.06	627	29.98	159	160	99.38
F3	1373	64.02	850	8.35	64	65	98.46
F4	328	92.68	250	3.20	7	7	100.00
F5	1292	93.73	1102	0.00	/	/	/

## Discussion

To our knowledge, we report the first GSS for *Ae. albopictus*. Tikok allows producing 97.8 % males following dieldrin treatment of third-instar larvae. Such a genetic tool may advantageously replace traditional sieving that has proven imperfect for this species [15, 16]. Tikok hence paves the way for the up scaling of incompatible/sterile insect techniques targeting *Ae. albopictus*. The measurement of some life history traits shows that the line can be easily maintained in the presence of dieldrin selection. However, Tikok still displays a number of drawbacks that need to be discussed and for which improvements must be considered.

The strong bias in sex ratio provided by Tikok could be considered as strong enough for sterile insect technique, although proper modelling will need to be carried out at each future environmental set-up chosen for pilot trials. In addition, since virtually all released females are vectors, the accidental release of females needs to be avoided in any case. Perfect sex separation is particularly critical for the incompatible insect technique [23] as released females will be fertile with incompatible males and may lead to the invasion of a hetereospecific *Wolbachia* in *Ae. albopictus* natural populations [5]. As shown by the characterization of surviving Tikok females following dieldrin selection, all tested females were heterozygous for *rdl*. In addition, these females were nearly fully fertile when crossed with non-translocated S-Run males. The recovery of a nearly perfect fecundity suggests that these females result from a meiotic recombination/translocation restoring chromosome integrity rather than from a recombination between the male locus and the linked *rdl*. Anyway, a good fitness of these RS Tikok females will undoubtedly provide any accidentally released females with good chances of laying viable eggs. As a consequence, R-Run translocation will have to be repeated as long as the obtained sex ratio distortion is not close to 100 %.

Alternatively, this genetic sexing tool could be used right away in a combined *Wolbachia*-irradiation strategy [4] where low irradiation doses allow sterilizing the few surviving and accidentally released Tikok females. First, we need to address whether Tikok line is suitable for mass rearing. Cage experiments in which selective pressure was relieved are informative in this respect. It appears that the dieldrin resistance conferring allele disappears from the population within 5 generations in the absence of selective pressure, suggesting that the dieldrin resistance conferring allele and/or its sex linkage have a strong genetic cost. We have shown that the < 2 % Tikok females surviving dieldrin selection are heterozygous and fully fertile, we propose that the rearrangement(s) leading to these few resistant females also lead to fully fertile sensitive males. In this case, such males would have an important fitness advantage over Tikok resistant males displaying affected fecundity (see Table 3). As a result, mass rearing of Tikok will require crossing GSS males with S-Run females every other generation. This would not necessarily need manual sex separation of S-Run females but instead crossing Tikok males with S-Run pupae collected 3 days after the first pupation, since any progeny originating from S-Run males accidentally released in the cage would be killed by dieldrin at larval stage. Lastly, provided Tikok is suitable for mass rearing, any release of irradiated and/or incompatible males will have to follow a thorough examination of mating competitiveness of released males. Indeed, it is absolutely required to demonstrate that the genetic background of Tikok does not alter male competitiveness.

The main drawback of this GSS is certainly the used marker, as dieldrin has been banned for decades in a number of countries. It must be emphasized that in no circumstances the insecticide should be used in the field. Larvae or even eggs [24] should be treated exclusively in the laboratory for sexing purposes. However, traces of dieldrin may persist in adults as previously reported in *Anopheles arabiensis* GSS [25]. Although the overall amount of dieldrin residues remaining in the mosquito body has been overestimated in previous studies [25], their main conclusions remain valid: sterile males released in the frame of SIT or other population suppression projects using rdl-based GSS would still carry dieldrin residues which can bio-accumulate in the environment and contribute to the increasingly worrying phenomena of insecticide resistance. This could be circumvented by different ways. First, dieldrin could be replaced by authorized pesticides, such as fipronil, for which cross-resistance with GABA receptor mutants has been previously reported [26–28]. Second, the present work shows the feasibility of genetic translocation for the generation of a sex separation tool in *Ae. albopictus*. The mutation conferring dieldrin resistance is thus far one of the few available robust selectable marker for this mosquito species. However, given the medical importance of this mosquito species, it is likely that other insecticide resistance markers (such as the recently reported *kdr* mutants [29] or carboxylesterase gene amplifications [30]) will be fully characterized in the next future and will be made available for the development of a GSS avoiding the use of banned insecticides. Lastly, sterile insect strategies are appealing as they are both highly specific and environmentally friendly. Therefore, a high throughput screen allowing the identification of “clean” markers may be a profitable investment. Temperature sensitive lethal mutations have been previously discovered for other Culicinae species [31] and used at industrial scales for the control of medflies [13, 32, 33]. Hence, the present work shows that the identification or the development of such a marker by random mutagenesis would pave the way to the implementation of fully clean sterile insect strategies that are urgently needed for the control of future emerging epidemics involving the tiger mosquito.

## Conclusions

We report the construction of the first Genetic Sexing Strain (GSS) for *Ae. albopictus*. This strain, obtained through the translocation of rdl^R^, conferring resistance to dieldrin, allows selecting 98% males following insecticide pressure on third instar larvae. Although Tikok appears to be easily maintained in insectaries provided insecticide pressure is maintained, this study stimulates further investigations allowing identifying “clean” markers that could be translocated following the methods described herein. Such a GSS will accelerate the implementation of environmental-friendly strategies aiming at controlling natural populations through mass release of sterilizing males, such as the Sterile or Incompatible Insect Techniques.
